# Sacrococcygeal epidural administration of 0.5% bupivacaine in seven cats undergoing pelvic or hind limb orthopaedic procedures

**DOI:** 10.1186/s13620-023-00231-2

**Published:** 2023-02-02

**Authors:** Xavier Torruella, Joanna Potter, Vilhelmiina Huuskonen

**Affiliations:** UCD Veterinary Hospital, School of Veterinary Medicine, University College Dublin, Belfield, Dublin, D04 W6F6 Ireland

**Keywords:** Caudal anaesthesia, Caudal epidural, Epidural anaesthesia, Epidural analgesia, Local anaesthesia

## Abstract

**Background:**

Epidural administration of local anaesthetic agents provides good intraoperative antinociception for orthopaedic procedures of the pelvis and the pelvic limb. However, in cats the spinal cord extends approximately to the level of the first sacrococcygeal vertebra, and therefore the sacrococcygeal epidural could be a safer alternative to the lumbosacral epidural in cats. This case series describes perioperative analgesia and the haemodynamic status of seven client-owned cats that received sacrococcygeal epidural injection of 0.5% bupivacaine and underwent orthopaedic hind leg or pelvic surgeries under general anaesthesia.

**Case presentation:**

Each cat received either 0.2 or 0.3 mL/kg of 0.5% bupivacaine with or without 0.2 mg/kg of morphine in the sacrococcygeal epidural space. Intraoperative antinociceptive response to surgical stimulus and haemodynamic changes were monitored and reported.

**Conclusion:**

In these seven anaesthetised cats, 0.2 or 0.3 mL/kg of 0.5% bupivacaine, administered alone or in combination with morphine into the sacrococcygeal epidural space, enhanced antinociception so that intraoperative rescue analgesia was unnecessary in all but one cat. It also reduced the anticipated requirement for postoperative opioid use. However, a high incidence of hypotension was observed in the cats in this report, and hence intraoperative blood pressure monitoring should be considered mandatory in anaesthetised cats following epidural injection of local anaesthetic agents, regardless of injection site.

## Background

Nociception caused by a surgical procedure may induce autonomic nervous system responses such as sympathetic stimulation [1]. To maintain the haemodynamic stability of the patient during general anaesthesia, undesirable autonomic responses should be avoided by providing adequate intraoperative antinociception.

Administration of a local anaesthetic in the lumbosacral epidural space is commonly performed in small animals for effective antinociception during hindlimb orthopaedic surgeries [2]. However, in cats the spinal cord ends approximately at the level of the first sacrococcygeal vertebra (S1) [3], leading to a higher lumbosacral epidural complication rate, such as piercing of meninges or subarachnoid injection, compared to dogs [2]. In addition, some pelvic fractures may render the anatomical landmarks difficult to identify, making the lumbosacral approach harder to perform. Because the cat spinal cord does not extend to the sacrococcygeal space, the sacrococcygeal epidural injection could be a safer alternative to the lumbosacral epidural in cats [4].

The sacrococcygeal epidural has been described for urethral catheterization in cats with urinary obstruction [5]. Otero et al. (2015) mention the use of sacrococcygeal epidurals for perineal or hind limb surgical procedures in cats, but they do not describe the perioperative analgesic or haemodynamic response [6]. To the authors’ knowledge, there are no publications which discuss the use of the sacrococcygeal epidural approach in the provision of antinociception in hind limb or pelvic orthopaedic procedures in cats. Our aim in this case series is to describe the perioperative analgesic and haemodynamic response to the sacrococcygeal epidural administration of 0.5% bupivacaine (with or without morphine), in seven client-owned cats undergoing orthopaedic hind leg or pelvic surgeries.

## Description of the cases

The seven cats described in this case report were referred to our hospital for orthopaedic procedures of the pelvis or the pelvic limb. Each cat received either 0.2 or 0.3 mL/kg of 0.5% bupivacaine^1^ with or without 0.2 mg/kg of morphine^2^ in the sacrococcygeal epidural space, using a hypodermic 25-gauge 16 mm needle, as described by O’Hearn et al. (2011). For the epidural injection the cat was positioned in sternal recumbency with the hind limbs extended caudally. The sacrococcygeal space was palpated between the sacrum and the first coccygeal vertebrae; moving the tail up and down helped with the identification of the correct space. The needle was advanced “blindly”, guided by the tactile sensation of ligament penetration. The block was performed by an anaesthesia resident with previous experience performing this technique (XT) or a board-certified veterinary anaesthesia diplomate (VH or JP).

Premedication and induction protocols are listed in Table 1. After induction of general anaesthesia, tracheal intubation was performed with a cuffed endotracheal tube (ETT), and anaesthesia was maintained with isoflurane^3^ or sevoflurane^4^ in 100% oxygen (O_2_). In each case, the concentration of the anaesthetic agent was adjusted as required (Table 2), based on anaesthetic depth and blood pressure. Spontaneous breathing was allowed but intermittent positive pressure ventilation^5^,^6^ (IPPV) was started if hypoventilation (end-tidal carbon dioxide (EtCO_2_) > 6.5 kPa) was observed, to maintain the EtCO_2_ within the normal range (4.5–6.0 kPa).

**Table 1:** General information and anaesthetic protocol in seven client-owned cats undergoing pelvic or hind limb orthopaedic procedures

Case	Case 1	Case 2	Case 3	Case 4	Case 5	Case 6	Case 7
Signalment	1-year-old DLH male (neutered)	1-year-old DSH male (neutered)	1-year-old DSH male (neutered)	1-year-old DSH female (spayed)	8-month-old DSH female (spayed)	10-year-old Norwegian Forest Cat female (spayed)	6-year-old British short hair female (spayed)
Body weight (kg)	2.5 kg	3.9 kg	4.9 kg	2.8 kg	1.9 kg	3.9 kg	3.2 kg
ASA Classification	ASA 3	ASA 2	ASA 2	ASA 2	ASA 2	ASA 3	ASA 2
Procedure	Hip fracture repair	MPL repair	Femoral fracture repair	Hip fracture repair	Left hind limb amputation	Left hind limb amputation (unresectable STS)	Hip luxation
Premedication drugs	Medetomidine^a^ (5 μg/kg IV) and methadone (0.3 mg/kg IV)	Midazolam^b^ (0.3 mg/kg IM), methadone (0.3 mg/kg IM), and alfaxalone^c^ (3 mg/kg IM)	Medetomidine (1 μg/kg IV) and methadone (0.3 mg/kg IV)	Medetomidine (5 μg/kg IM), methadone (0.3 mg/kg IM), and alfaxalone (3 mg/kg IM)	Midazolam (0.3 mg/kg IM), methadone (0.3 mg/kg IM), and alfaxalone (5 mg/kg IM)	Medetomidine (5 μg/kg IM), methadone (0.3 mg/kg IM), and ketamine^d^ (2 mg/kg IM)	Medetomidine (4 μg/kg IV) and methadone (0.2 mg/kg IV)
Induction drugs	Propofol^e^ (2 mg/kg IV) and ketamine (2 mg/kg IV)	Propofol (2 mg/kg IV) and ketamine (2 mg/kg IV)	Alfaxalone (1 mg/kg IV) and ketamine (2 mg/kg IV)	Propofol (2 mg/kg IV)	Alfaxalone (1 mg/kg IV) and ketamine (2 mg/kg IV)	Alfaxalone (1 mg/kg IV)	Alfaxalone (2 mg/kg IV)
Maintenance agent	Sevoflurane	Sevoflurane	Sevoflurane	Isoflurane	Isoflurane	Isoflurane	Isoflurane
Breathing system and fresh gas flow	Paediatric circle^f^ 0.5 L/min	Mini-Lack^g^ 0.8 L/min	Mini-Lack 0.8 L/min	Paediatric circle 0.8 L/min	Paediatric t-piece^h^ 0.5-2 L/min	Paediatric circle 0.8 L/min	Mini-Lack 0.5 L/min
IPPV	Yes	No	No	Yes	Yes	No	No

*ASA* American Association of Anesthesiologists, *DLH* domestic longhair cat, *DSH* domestic shorthair cat, *IPPV* intermittent positive pressure ventilation, *MPL* medial patellar luxation, *STS* soft tissue sarcoma

^a^Medetomidine hydrochloride: Domitor 1 mg/mL, Duggan Veterinary, Ireland

^b^Midazolam: Hypnovel® 5 mg/mL, Roche Products Ltd., UK

^c^Alfaxalone: Alfaxan® 10 mg/mL, Jurox Ltd., Ireland

^d^Ketamine: Ketomidor 100 mg/mL, Chanelle pharmaceuticals, Ireland

^e^Propofol: Propofol-Lipuro 1%, B. Braun Medical Inc., Ireland

^f^Circle breathing system: Infant rebreathing limbs, Intersurgical, Ltd., UK

^g^Mini-Lack nonrebreathing system: Mini-Lack Anaesthetic Breathing System, Burtons Medical Equipment, UK

^h^Paediatric T-piece nonrebreathing system: Infant T-piece breathing system, Intersurgical, Ltd., UK

**Table 2:** Analgesic protocol and intraoperative complications in seven client-owned cats undergoing pelvic or hind limb orthopaedic procedures

**Case**	Case 1	Case 2	Case 3	Case 4	Case 5	Case 6	Case 7
Sacrococcygeal epidural	0.5% bupivacaine (0.2 mL/kg)	0.5% bupivacaine (0.3 mL/kg)	0.5% bupivacaine (0.2 mL/kg)	0.5% Bupivacaine (0.2 mL/kg)	0.5% bupivacaine (0.2 mL/kg)	0.5% bupivacaine (0.2 mL/kg) and morphine (0.2 mg/kg)	0.5% bupivacaine (0.2 mL/kg) and morphine (0.2 mg/kg)
EtAA (%)	EtSevo 1.4–2.2	EtSevo 1.4–2.0	EtSevo 1.3–1.5	EtIso 1.1–1.3	EtIso 1.1–1.3	EtIso 0.9–1.2	EtIso 0.9–1.2
Additional intraoperative analgesia	None	None	None	Two boluses of ketamine (0.5 mg/kg IV)	Intraneural infiltration of femoral and sciatic nerves with 2% lidocaine	Intraneural infiltration of femoral and sciatic nerves with 2% lidocaine	None
Postoperative NSAID	Meloxicam 0.05 mg/kg IV q 24 h	Meloxicam 0.1 mg/kg IV followed by 0.05 mg/kg IV q 24 h	Meloxicam 0.05 mg/kg IV q 24 h	Meloxicam 0.05 mg/kg IV q 24 h	Meloxicam 0.1 mg/kg IV followed by 0.05 mg/kg IV q 24 h	Meloxicam 0.1 mg/kg IV followed by 0.05 mg/kg IV q 24 h	Meloxicam 0.05 mg/kg IV q 24 h
Postoperative GCMPS before administration of additional opioid analgesia	Not performed	0/20	4/20	Not performed	Not performed	2/20	6/20
Additional postoperative opioid analgesia	Methadone 0.2 mg/kg IV q 6 h	Methadone 0.2 mg/kg IV followed by buprenorphine 10 μg/kg q 6 h	Methadone 0.2 mg/kg IV followed by buprenorphine 10 μg/kg q 6 h	Methadone 0.2 mg/kg IV q 6 h	Methadone 0.2 mg/kg IV q 4 h	Buprenorphine 15 μg/kg q 6 h	Methadone 0.2 mg/kg IV q 4 h
Time of first dose of additional postoperative opioid analgesia	6 h after epidural	6 h after epidural	10 h after epidural	8 h after epidural	8 h after epidural	24 h after epidural	11 h after epidural
Intraoperative complications	Bradycardia, hypotension, and hypothermia	Hypotension and hypothermia	Hypotension	Bradycardia, hypotension, and hypothermia	Bradycardia, hypotension, and hypothermia	Bradycardia, hypotension, and hypothermia	Bradycardia, hypotension, and hypothermia

*EtAA* end-tidal anaesthetic agent, *EtIso* end-tidal isoflurane concentration, *EtSevo* end-tidal sevoflurane concentration, *GCMPS* Glasgow Composite Measure Pain Scale-feline, *NSAID* nonsteroidal anti-inflammatory drug

Intravenous fluids^7^ were administered at a rate of 5 mL/kg/h throughout the procedure for each cat. Depth of anaesthesia was assessed by subjective methods: palpebral reflex, jaw tone, eye position and response to surgical stimuli. The EtCO_2_, end-tidal anaesthetic agent (EtAA), non-invasive blood pressure (NIBP), respiratory rate, heart rate (HR) and rhythm (ECG), haemoglobin oxygen saturation (SpO_2_) and oesophageal temperature were monitored in all patients, either with a multiparameter monitor,^8^ or with a combination of multiparameter monitor, portable NIBP monitor^9^ and portable SpO_2_ monitor.^10^ Hypotension was defined as a mean arterial pressure (MAP) ≤ 65 mmHg. Active warming was provided throughout general anaesthesia with a forced air warming blanket.^11^ If the cats were hypothermic postoperatively, they were recovered in a paediatric incubator^12^ controlled to maintain an environment of 40% O_2_, air temperature of 37ºC and humidity of 35%.

Intraoperative rescue analgesia was administered if the HR or MAP increased > 20% from the baseline values in response to surgical stimulus. Each cat received meloxicam^13^ at the end of anaesthesia, and either methadone^14^ or buprenorphine^15^ postoperatively. In cases 2, 3, 6 and 7 postoperative pain was assessed every four hours with the Glasgow Feline Composite Measure Pain Scale (GCMPS-feline) to determine the need for additional analgesia. When the pain score was not assessed, postoperative analgesia was administered 6–8 h after epidural administration, i.e., when the effect of the local anaesthetic was expected to end. The details of the case management are summarized in Tables 1 and 2.

### Case 1

Ten minutes after the sacrococcygeal epidural injection of bupivacaine, the cat’s MAP decreased from 90 mmHg to < 60 mmHg following a drop in HR from 110 beats per minute (bpm) to 90 bpm. Glycopyrronium bromide^16^ (10 µg/kg) was administered IV to increase the heart rate and blood pressure. This was initially successful, but 20 min after the start of surgery, the cat’s HR and MAP decreased to 105 bpm and 62 mmHg, respectively. A slow IV bolus of ephedrine^17^ (0.2 mg/kg) was required to increase the MAP which was then maintained between 70 and 80 mmHg. After this episode, the haemodynamic parameters remained stable. The cat was also hypothermic intraoperatively (35.5 ºC), despite active warming with a forced air warming blanket. No nociception was observed in response to surgical stimulation. The cat was recovered from anaesthesia in a paediatric incubator due to hypothermia.

### Case 2

Fifteen minutes after the epidural injection of bupivacaine, the cat’s MAP decreased from 70 to 65 mmHg, and a slow IV bolus of ephedrine (0.1 mg/kg) was administered. Thirty minutes later, the MAP decreased to 62 mmHg. A second dose of ephedrine (0.1 mg/kg) increased the MAP above 70 mmHg until the end of surgery. No nociception was observed in response to surgical stimuli. Recovery from anaesthesia was smooth. Due to hypothermia (34.6ºC), the cat was recovered in a paediatric incubator.

### Case 3

Prior to the epidural injection, the cat’s HR was 150 bpm and MAP 72 mmHg. Ten minutes after the epidural administration of bupivacaine, the HR remained at 130 bpm but the MAP decreased to 55 mmHg. The hypotension was treated with a single slow IV bolus of ephedrine (0.1 mg/kg), which increased the MAP and maintained it above 65 mmHg until the end of anaesthesia. No sympathetic responses were recorded during the surgical stimuli and recovery from anaesthesia was smooth.

### Case 4

Before the epidural administration of bupivacaine, the cat’s HR was 85 bpm and MAP 78 mmHg. Five minutes after the epidural injection the MAP dropped to 59 mmHg. One dose of glycopyrronium bromide (10 µg/kg) was administered IV to increase the HR above 100 bpm and MAP above 60 mmHg. During reduction of the pelvic fracture, an increase in MAP from 60 to 80 mmHg and an increase in HR from 130 to 160 bpm was observed, and an IV bolus of ketamine (0.5 mg/kg) was administered to provide rescue analgesia. A second bolus of ketamine (0.5 mg/kg) was administered IV during suturing of skin due to a similar increase in HR and MAP. On recovery from anaesthesia the cat was hypothermic with a rectal temperature of 35 ºC, and it was recovered in a paediatric incubator.

### Case 5

During preparation of the surgical area the cat’s MAP was 68 mmHg with a HR of 150 bpm. Five minutes after the epidural injection of bupivacaine, the MAP decreased to 59 mmHg and the HR to 120 bpm. Atropine^18^ (20 µg/kg) was administered IV which increased the HR to 160 bpm. With the increase in HR, the MAP initially increased to 72 mmHg, but then decreased again below 60 mmHg, and a 3 mL/kg bolus of colloid (succinylated gelatin)^19^ was administered IV. Despite the colloid bolus, the MAP did not improve. The cat was moved to the operation theatre where a dobutamine^20^ infusion was started with a variable rate of 2–4 µg/kg/min to maintain MAP above 65 mmHg. When an intraneural infiltration of the sciatic and femoral nerves with 1 mL of 2% lidocaine^21^ was performed before cutting both nerves, an increase in HR from 120 to 150 bpm was observed. No other sympathetic responses to surgical stimuli were observed during the surgical procedure. Intraoperatively the cat was mildly hypothermic (35.8ºC) but became normothermic before recovery. Recovery from anaesthesia was smooth.

### Case 6

Five minutes after the epidural injection of bupivacaine and morphine, the cat’s HR decreased from 110 to 95 bpm and the MAP decreased from 80 to 64 mmHg. A dose of glycopyrronium bromide (10 µg/kg IV) was administered after which the HR was maintained around 120 bpm and the MAP above 65 mmHg until the end of the procedure. Unlike in case 5, no sympathetic responses were observed during the intraneural infiltration of the sciatic and femoral nerves or during the surgical procedure. The cat was recovered from anaesthesia in a paediatric incubator due to postoperative hypothermia (35.1ºC). The recovery was smooth.

### Case 7

Twenty minutes after the epidural injection, a decrease in HR from 130 to 100 bpm was observed, and the MAP decreased from 78 to 60 mmHg. A dose of glycopyrronium bromide (10 µg/kg IV) was administered, which initially increased the HR to 120 bpm and MAP to 75 mmHg. Thereafter MAP gradually decreased but was maintained above 65 mmHg until the end of the procedure. No sympathetic responses to surgical stimuli were observed. The cat was recovered in a paediatric incubator due to hypothermia (35.4ºC).

## Discussion

When starting to write this case series, our main aim was to report perioperative analgesia provided by sacrococcygeal epidural administration of 0.5% bupivacaine (with or without morphine) in client-owned cats undergoing orthopaedic pelvic or pelvic limb surgeries. A secondary aim was to give a description of the haemodynamic effects of the block; however, due to the lack of a control group, we cannot conclude that the haemodynamic changes described were caused solely by the epidural administration.

We used the “blind” technique described by O`Hearn et al. (2011) to perform the sacrococcygeal epidural injections [4], although electrolocation is considered a superior method to identify the epidural space [6]. The electrolocation technique involves the use of neurostimulation to elicit lateral twitching of the tail to confirm the correct needle placement [6]. The rationale for the use of the “blind” technique was that it does not require specialist training or equipment, and therefore it could reasonably be used also in the general practice setting.

The lumbosacral plexus originates from the L3-S3 spinal nerves and is responsible for the innervation of the hind limbs in dogs and cats [3, 7]. The volume of local anaesthetic described in the literature for lumbosacral epidural injection in cats undergoing hind leg orthopaedic surgeries is 0.2 mL/kg [2]. When 0.2 mL/kg of methylene blue is injected into the lumbosacral epidural space in cats, it is distributed up to the level of spinal nerves arising from L1-L2 [8]. The volume of 0.5% bupivacaine used in Cases 1, 3, 4, 5, 6 and 7 was also 0.2 mL/kg; this is the volume described by O`Hearn et al. (2011) for urethral catheterisation when injected into the sacrococcygeal space in cats [4]. We were initially worried that this volume might not be sufficient to distribute the local anaesthetic to the desired (L3) spinal nerve level to allow a hind limb orthopaedic procedure when injected into the sacrococcygeal space. Therefore, in Case 2 the volume of 0.5% bupivacaine administered was 0.3 mL/kg, which is the volume used by Otero et al. (2015) for sacrococcygeal epidurals for perineal or hind limb surgical procedures in cats [6]. In contrast, a study by Pratt et al. (2020) described the use of 0.22 mg/kg of 0.5% bupivacaine (with or without morphine 0.1 mg/kg) administered into the sacrococcygeal epidural space in cats with urinary obstruction; this dose of bupivacaine corresponds to a volume of 0.044 mL/kg which is much lower than the volume used in our cats [5]. Pratt et al. (2020) found that with this dose, the anaesthetic and analgesic requirements during urethral catheterization were decreased without any negative haemodynamic changes [5]. However, further studies need to be conducted to evaluate the extent of the epidural spread of the local anaesthetic when this lower volume is used via the sacrococcygeal route for pelvic limb orthopaedic procedures. Based on the available literature, we elected to continue using 0.2 mL/kg of 0.5% bupivacaine in the rest of the enrolled cats.

Although it is important to administer a sufficient volume of the local anaesthetic to ensure that the necessary structures are desensitised, a potential downside is extension of the sympathetic block. Epidural administration of local anaesthetics may produce hypotension by blocking preganglionic sympathetic efferent fibres, inducing vasodilation in the blocked areas [9]. The sympathetic trunk receives its preganglionic efferent fibres from T1 to L2-L3; therefore, the extent of sympathetic block will depend on the volume of local anaesthetic administered and whether the spread of the agent is sufficient to block some of these preganglionic fibres [9]. With the volumes used in this report, the preganglionic sympathetic efferent fibres should not have been fully compromised [8, 9]. Nevertheless, the effect of blockade of these sympathetic efferent fibres can become more evident when combined with the vasodilatory effects of volatile anaesthetic agents. Additionally, reduced or absent nociception as a result of successful epidural may also lead to a reduced level of sympathetic tone, thus compounding the hypotension arising from such sympathetic blockade. Intraoperative hypotension (MAP ≤ 65 mmHg) was observed in all seven enrolled cats, between five and 20 min after sacrococcygeal epidural injection of 0.5% bupivacaine, requiring interventions such as reducing the dose of inhalant anaesthetic agent, administering anticholinergic drugs or ephedrine, bolusing crystalloid or colloid fluids, or starting a positive inotrope (dobutamine) infusion. We cannot be certain whether the extent of distribution of bupivacaine in Case 2 made this cat more refractory to treatment of hypotension ultimately requiring two doses of glycopyrronium bromide and two doses of ephedrine before the blood pressure was adequately restored.

A systematic review of isoflurane and sevoflurane minimum alveolar concentration (MAC) in domestic cats by Shaugnnessy and Hofmeister (2014) reflected that the MAC of isoflurane can vary from 1.2 to 2.22% and the MAC of sevoflurane from 2.5 to 3.95% [10]. In contrast with the systematic review, the ranges of EtIso and EtSevo observed in our cases were 0.9–1.3% and 1.3–2.2%, respectively, suggesting a reduction in the volatile agent requirement. Similar results were found by Troncy et al. (2002): they observed a decrease in isoflurane requirement in cats undergoing surgical procedures that had received bupivacaine and morphine in the lumbosacral epidural space preoperatively [11]. The MAC of an anaesthetic agent is defined as the alveolar concentration of the inhaled anaesthetic at which 50% of animals do not move in response to a surgical stimulus [12] and is the standard for comparison of volatile anaesthetic potency [12]. Decreasing the MAC of an inhalant anaesthetic agent is beneficial for the patient because it allows a lower dose of the anaesthetic to be administered, thereby decreasing its negative effects, such as vasodilation, decreased myocardial contractility and respiratory depression, which are dose-dependent [13]. The more intense the nociceptive stimulus, the higher the dose of inhalant anaesthetic required to maintain an adequate depth of anaesthesia to blunt the responses to such nociception. The higher the dose of the inhalant anaesthetic, the more pronounced are its negative effects, particularly hypotension. Therefore, although epidural administration of local anaesthetics may cause a mild hypotension, the enhanced intraoperative antinociception cannot be overstated – ultimately this is likely to lead to improved overall haemodynamic stability when compared to animals who did not receive appropriate antinociception and improved postoperative pain control. It is important to stress the necessity of analgesic techniques, particularly from an ethical perspective, and encourage their routine use.

In the enrolled cats, intraoperative rescue analgesia was administered if the HR or MAP increased > 20% from the baseline in response to surgical stimulus. In Cases 1 and 4, pelvic fracture repairs were performed. Case 1 did not need any rescue analgesia due to the absence of nociception, but Case 4 received two 0.5 mg/kg boluses of ketamine IV, one during fracture reduction and one during skin suturing. This could potentially be explained by surgical stimulation in an area more cranial than that achieved by the epidural distribution, or in dermatomes that are supplied by nerves more cranial than that achieved by the epidural. Cases 2 and 3 did not require intraoperative rescue analgesia during their fracture repairs. Cases 5 and 6 were anaesthetised for hind limb amputation and no intraoperative rescue analgesia was needed; however, in both cases the surgeons administered 2% lidocaine intraneurally into the femoral and sciatic nerves before resection that could have prevented potential intraoperative nociception. If the epidural is deemed successful, intraneural lidocaine administration should be unnecessary. Case 7 required no rescue analgesia during its hip luxation repair. Similar results were observed by Ferrero et al. (2021), when they retrospectively compared preoperative lumbosacral epidural administration of local anaesthetic with two different loco-regional anaesthetic techniques in dogs undergoing pelvic limb surgeries; only 18.8% of the dogs needed intraoperative analgesia when epidural administration of a local anaesthetic was successfully performed [14].

All cats received meloxicam at the end of the procedure. In four cats the GCMPS-feline was assessed every four hours after recovery from anaesthesia to assess the requirement for opioid analgesia post-epidural administration. Our cut-off value for administration of additional postoperative opioid analgesia was ≥ 5/20, as described by Calvo et al. (2014) and Reid et al. (2017) [15, 16]. All pain scoring was performed by trained personnel such as veterinary nurses or veterinary interns. Cases 2, 3 and 6 received postoperative opioid analgesia even though their GCMPS-feline scores were 0/20, 4/20 and 2/20, respectively, due to the assessor’s perception that the patient was painful regardless of the pain score. The postoperative pain scores were not assessed in Cases 1, 4 and 5. Case 1 received postoperative opioid analgesia six hours after the epidural, and Cases 4 and 5 eight hours after the epidural, i.e., at the time when the sensory block provided by bupivacaine was expected to wane.

The duration of sensory block after lumbosacral epidural administration of 0.5% bupivacaine alone (i.e., without opioids) has been described to last between six to eight hours in dogs and cats [2, 17]. Epidural administration of morphine together with bupivacaine is known to increase the duration of action of bupivacaine and enhance the analgesic effect of epidural bupivacaine in both dog and cats. [2, 11]. The most common adverse effect of epidural morphine is urinary retention [2, 11, 14], but no urinary retention was observed in Cases 6 and 7. A retrospective study in dogs, in which lumbosacral epidural administration of local anaesthetics was compared with other locoregional techniques, found that the time to first postoperative methadone dose after epidural administration of bupivacaine together with morphine was approximately eight hours; they also found that 88.1% of the dogs in the epidural group required postoperative analgesia [14], suggesting that while postoperative opioid consumption is decreased when epidural bupivacaine is combined with morphine for hind limb orthopaedic surgeries, it is still necessary.

Lumbosacral epidural injections in cats are associated with complications such as piercing of the meninges, leakage of the CSF, and venous plexus puncture [2]. This is due to the length of the feline spinal cord, which ends between L7 and S3 [3]. Given the lack of these complications in the cases described here, we suggest that in feline hind limb or pelvic orthopaedic procedures the sacrococcygeal technique is safer when compared with the lumbosacral technique. However, intraoperative hypotension was observed in all cases, which is also commonly described after lumbosacral epidural injection of local anaesthetics in cats and dogs [2, 11, 14]. The hypotension was likely caused by a combination of factors, namely inhalant anaesthetic agents, epidural bupivacaine, bradycardia and hypothermia. Therefore, it is very important that blood pressure is monitored, and active warming methods used, and that the operator is prepared to treat hypotension in anaesthetised cats after epidural administration of local anaesthetic agents, regardless of the dose or route used. A detailed comparison of the two epidural techniques, lumbosacral and sacrococcygeal, and their effects on haemodynamic stability during surgery would be welcomed.

A limitation of this case report is that not all cases were performed by the same anaesthetist, introducing variables such as different anaesthetic protocols, different approaches to the treatment of intraoperative bradycardia or hypotension, and different postoperative analgesic protocols. By implementing the same anaesthetic protocol, the same approach to haemodynamic changes and the same postoperative analgesia protocol, we could have decreased the number of variables and made evaluation of perioperative analgesia easier. However, in routine clinical practice there will be situations where there are multiple anaesthetists or clinicians in charge of the anaesthetic, and we wanted to mimic the clinical setting rather than a research setting. Another limitation is the lack of a control group: it is possible that the sacrococcygeal epidural administration of local anaesthetics contributed to the intraoperative hypotension, but without a control group it cannot be verified. A further limitation in our case series is the lack of consistent pain scoring postoperatively in all cases. Unfortunately, our hospital suffered staffing shortages as a result of the global pandemic, and subsequently, postoperative case management involved timed administration of analgesics rather than administration in response to a pain score intervention.

## Conclusions

In these seven cats, 0.2 or 0.3 mL/kg of 0.5% bupivacaine administered alone or in combination with morphine into the sacrococcygeal epidural space enhanced antinociception so that intraoperative rescue analgesia was unnecessary in all but one cat. It also reduced the anticipated requirement for postoperative opioid use. However, similar to lumbosacral epidural, a high incidence of hypotension was observed in the enrolled cats, and therefore intraoperative blood pressure and heart rate monitoring should be considered mandatory in anaesthetised cats following epidural injection of local anaesthetic agents, regardless of injection site.

## Data Availability

The data used are available from corresponding author on reasonable request.

## Abbreviations

ASA: American Association of Anesthesiologists
CO: Cardiac output
CSF: Cerebrospinal fluid
DLH: Domestic longhair cat
DSH: Domestic shorthair cat
ECG: Electrocardiography
EtAA: End-tidal anaesthetic agent concentration
EtCO_2_: End-tidal carbon dioxide concentration
EtIso: End-tidal isoflurane concentration
EtSevo: End-tidal sevoflurane concentration
ETT: Endotracheal tube
GCMPS-feline: Glasgow Feline Composite Measure Pain Scale
HR: Heart rate
IM: Intramuscular
IPPV: Intermittent positive pressure ventilation
IV: Intravenous
MAC: Minimum alveolar concentration
MAP: Mean arterial pressure
MPL: Medial patellar luxation
NIBP: Non-invasive blood pressure
NSAID: Nonsteroidal anti-inflammatory drug
O_2_: Oxygen
RR: Respiratory rate
S1: First sacrococcygeal vertebra
SpO_2_: Haemoglobin oxygen saturation
STS: Soft tissue sarcoma
SVR: Systemic vascular resistance

## Footnotes

1 Bupivacaine: Marcain 0.5%, Polyamp SteriPack, Aspen Pharmacare, Ireland.

2 Morphine: Morphine sulphate 10 mg/ml, Mercury Pharma, Ireland.

3 Isoflurane: IsoFlo, Zoetis, Belgium.

4 Sevoflurane: SevoFlo, Zoetis, Belgium.

5 Circle breathing system ventilator: Datex Ohmeda Excel 210 SE anaesthesia machine, 7900 smart ventilator, Datex, USA.

6 T-piece breathing system ventilator: Small Animal Ventilator SAV03, Vetronic, UK.

7 Hartmann’s solution: Aquapharm 11 Hartmann’s solution for infusion, Duggan Veterinary, Ireland.

8 Multiparameter anaesthetic monitor: B40 patient monitor, GE Healthcare, Ireland.

9 Portable oscillometric blood pressure monitor: Vet20, Suntech, USA.

10 Portable pulse oximeter: LifeVet PT, Eickemeyer, Germany.

11 Warming device: 3 M Bair Hugger® Warming Units, Canada.

12 Paediatric incubator: Hill-Rom Air-Shields, Hillenbrand Industry, USA.

13 Meloxicam: Loxicom 5 mg/mL, Norbrook Laboratories Ltd., UK.

14 Methadone: Synthadon 10 mg/mL, Animalcare, UK.

15 Buprenorphine: Bupaq 0.3 mg/mL, Chanelle pharmaceuticals, Ireland.

16 Glycopyrrolate: Glycopyrronium bromide 0.2 mg/mL, Mercury Pharma, Ireland.

17 Ephedrine: Ephedrine hydrochloride 30 mg/mL, Ethypharm, France.

18 Atropine: Atropine sulfate 600 mg/mL, Hameln Pharma, UK.

19 Succinylated gelatin: Gelofusin 40 mg/mL, B. Braun, Germany.

20 Dobutamine: Dobutamine 12.5 mg/mL, Mercury Pharma, Ireland.

21 Lidocaine: Lidocaine hydrochloride 2%, B. Braun Medical Ltd., Ireland.
